# Dynamic Microtubules Promote Synaptic NMDA Receptor-Dependent Spine Enlargement

**DOI:** 10.1371/journal.pone.0027688

**Published:** 2011-11-11

**Authors:** Elliott B. Merriam, Derek C. Lumbard, Chris Viesselmann, Jason Ballweg, Matthew Stevenson, Lauren Pietila, Xindao Hu, Erik W. Dent

**Affiliations:** 1 Neuroscience Training Program, University of Wisconsin School of Medicine and Public Health, Madison, Wisconsin, United States of America; 2 Department of Neuroscience, University of Wisconsin School of Medicine and Public Health, Madison, Wisconsin, United States of America; Federal University of Rio de Janeiro, Brazil

## Abstract

Most excitatory synaptic terminals in the brain impinge on dendritic spines. We and others have recently shown that dynamic microtubules (MTs) enter spines from the dendritic shaft. However, a direct role for MTs in long-lasting spine plasticity has yet to be demonstrated and it remains unclear whether MT-spine invasions are directly influenced by synaptic activity. Lasting changes in spine morphology and synaptic strength can be triggered by activation of synaptic NMDA receptors (NMDARs) and are associated with learning and memory processes. To determine whether MTs are involved in NMDAR-dependent spine plasticity, we imaged MT dynamics and spine morphology in live mouse hippocampal pyramidal neurons before and after acute activation of synaptic NMDARs. Synaptic NMDAR activation promoted MT-spine invasions and lasting increases in spine size, with invaded spines exhibiting significantly faster and more growth than non-invaded spines. Even individual MT invasions triggered rapid increases in spine size that persisted longer following NMDAR activation. Inhibition of either NMDARs or dynamic MTs blocked NMDAR-dependent spine growth. Together these results demonstrate for the first time that MT-spine invasions are positively regulated by signaling through synaptic NMDARs, and contribute to long-lasting structural changes in targeted spines.

## Introduction

Dendritic spines of excitatory central nervous system (CNS) neurons have been studied extensively because of their importance in synaptic plasticity, learning and memory. Elegant studies in living mice have demonstrated that many spines are stable throughout the life of an animal, while others change shape and size [1], [2]. Subsequent experiments using similar paradigms have shown that even in adulthood structural plasticity of spines can occur and is important for encoding sensory and motor memories [3], [4], [5]. Although not imaged directly in a living brain, other studies have demonstrated that much of this structural plasticity in dendritic spines occurs through the dynamic reorganization of actin filaments [6], [7], [8]. However, recent studies from our lab and others indicate that microtubules (MTs) are also capable of transiently entering dendritic spines [9], [10], [11], [12] and interacting with the actin cytoskeleton [11].

MT invasions of spines are associated with neuronal depolarization [10], but it is unclear whether these invasions occur downstream of local signaling through synaptic glutamate receptors. It is also unclear whether MTs contribute to long-term structural or functional changes in the spines they enter. Furthermore, it is not known whether MTs contribute directly to long-lasting structural changes in spines [13].

Here we report that acute activation of synaptic NMDARs, a crucial determinant of long-term synaptic potentiation [14], triggers an increase in the frequency of MT-spine invasions. We also show that NMDAR-dependent increases in spine size are substantially larger in spines targeted by MTs, and that individual MT invasions are associated with rapid spine enlargement. Together these data demonstrate conclusively that MTs are playing a major, and heretofore unknown, role in NMDAR-dependent spine plasticity.

## Results

### Increased frequency of MT-spine invasions following acute synaptic NMDAR activation

**Figure 1:**
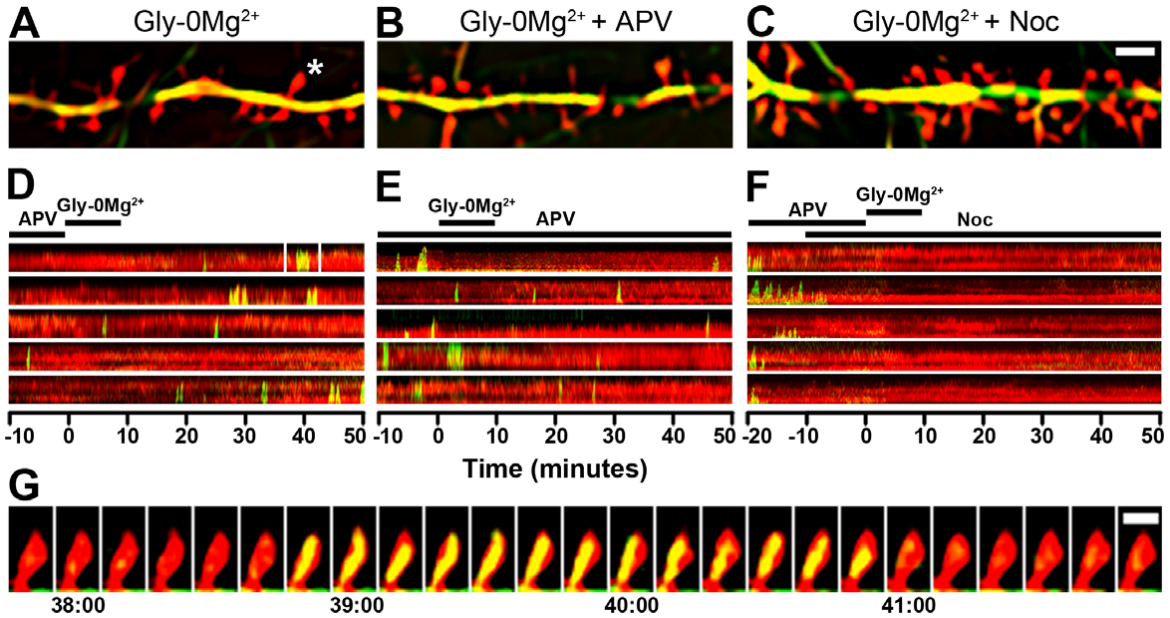
Acute activation of synaptic NMDARs promotes MT-spine invasions. (A - C) Total internal reflection fluorescence microscopy (TIRFM) images of dendrites from cultured hippocampal neurons transfected with DsRed2 (red) and EGFP-α-tubulin (green) and treated with glycine in 0Mg^2+^ solution (A) with additional APV (B) or nocodazole (C). Spine labeled with “*” in (A) is depicted in the top kymograph of (D) and again in (G). Scale bar, 3 µm. (D - F) Kymographs depicting transient entry of microtubules (MTs) into individual dendritic spines from the cells shown in A - C (respectively). Experimental paradigm of each experimental group is shown above the top kymograph. Top kymograph in (D) corresponds to labeled spine in (A). The invasion shown in the boxed region of (D) is depicted in (G). (G) Sequential frames show a MT entering the labeled spine from (A) and (D). The MT enters at t = 38∶50 (28 min and 50 sec after treatment with Gly-0Mg^2+^), and remains in the spine for 2 minutes before exiting. Scale bar, 1 µm.

Previous studies have shown that dynamic microtubule (MT) entry into dendritic spines can be promoted with neuronal depolarization [10] and that pharmacological inhibition of MT dynamics blocks long-term potentiation (LTP) in hippocampal slices [11]. Based on these results, we hypothesized that MT entry into spines may directly contribute to NMDAR-dependent plasticity at the level of individual spines. To test this, we used two-color total internal reflection fluorescence microscopy (TIRFM) to image 20–27DIV mouse hippocampal neurons co-transfected with EGFP-α-tubulin (to label MTs) and DsRed2 (to label cell volume) at 10 second intervals for 60–70 minutes (Fig. 1). To rapidly activate synaptic NMDARs [15], [16], [17] we pre-incubated cultured neurons with the NMDAR antagonist D,L-APV (200 µM) starting 16–24 hours prior to imaging and imaged cells in APV for 10 minutes before replacing it with 200 µM glycine in a 0 mM MgCl_2_ solution for 10 minutes, followed by washout (Fig. 1D).

**Figure 2:**
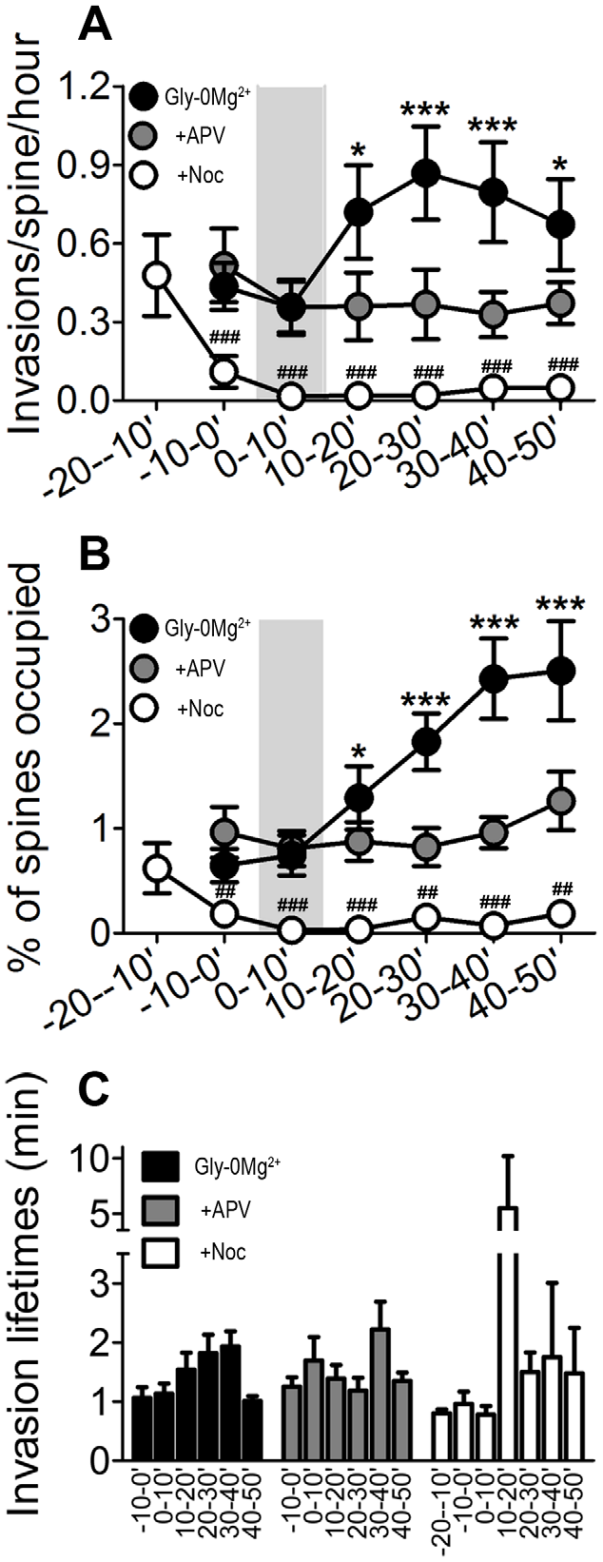
MT-spine invasions occur more frequently and occupy a larger percentage of spines after synaptic NMDAR activation. (A) Effect of NMDAR activation on MT-spine invasion frequencies. Data are binned into 10-minute intervals to assess changes in invasion frequencies over time within each experimental group. Light grey region indicates the timing of Gly-0Mg^2+^ treatment. Effects of time and treatment condition were assessed with a two-way ANOVA with repeated measures and Bonferonni post-test to compare time columns (* p<0.05, *** p<0.001). Effect of nocodazole treatment on MT invasion frequency (independent of NMDAR activation) was assessed with a one-way ANOVA with repeated measures and Dunnett's post-test (# p<0.05, ## p<0.01, ### p<0.001). (B) Effects of Gly-0Mg^2+^ treatment on the average percent of spines occupied by MTs at each time frame. Data binned and analyzed as in (A). (C) MT-spine invasion lifetimes before, during, and after NMDAR activation. Data are binned into 10-minute intervals. Invasion lifetimes did not change significantly over time in any of the experimental conditions and did not differ between conditions (two-way ANOVA). Of the few invasions that occurred in the presence of 200 nM nocodazole, some persisted in spines for a long duration (>5 min), but this effect was not significant. All graphs show mean ± SEM.

Following synaptic NMDAR activation, the frequency of MT-spine invasions increased by 75%, from 0.44±0.09 to 0.76±0.16 (mean ± SEM) invasions/spine/hour (n = 9 cells, 1115 spines, 5 preparations) (Fig. 1D,G and 2A). Synaptic NMDAR activation also increased the average percent of spines that were occupied by MTs in each imaging frame after the treatment (Fig. 2B). Overall, the average percent of spines occupied by MTs in each frame tripled from 0.65±0.16 before NMDAR activation to 1.99±0.33 (mean ± SEM) after activation (Fig. 2B). The total % of spines invaded across all cells treated with Gly-0Mg^2+^ was 21%. We also examined the effects of NMDAR activation on the amount of time each invading MT spent in a spine after entering (invasion lifetime). Invasion lifetimes after synaptic NMDAR activation were not significantly different from baseline levels (Fig. 2C). Thus, synaptic NMDAR activation specifically affects MT invasion frequency, resulting in a higher percentage of spines occupied by MTs following activation.

To confirm that this increase in MT invasions was dependent on NMDARs, we maintained some cells in APV throughout the experiment. No changes in MT-invasion frequency or lifetime, or the percent of spines occupied, were observed when cells were maintained in APV (n = 9 cells, 1310 spines, 4 preparations) (Fig. 1B,E and 2A-C). The total % of spines invaded across all cells treated with Gly-0Mg^2+^ in the presence of APV was 11%. Thus, the increases in MT-spine invasions we observed were NMDAR-dependent. To determine whether APV withdrawal alone is sufficient to increase MT-spine invasions, we pre-incubated other cells in APV and imaged them during washout with normal ECS. No changes in MT-invasion frequency or lifetime were observed when cells underwent APV withdrawal alone (data not shown).

To determine if MT dynamic instability, the stochastic elongation and shrinkage of MT plus ends, is required for MT spine invasions, we suppressed MT dynamics before, during and after synaptic NMDAR activation by pretreating neurons with a low dose (200 nM) of the MT-destabilizing drug nocodazole 10 minutes before synaptic NMDAR activation. At this dose nocodazole treatment did not trigger MT depolymerization [11], but did inhibit basal MT-spine invasions and also abolished NMDAR-dependent increases in MT-spine invasions (n = 6 cells, 881 spines, 4 preparations) (Fig. 1C,F and 2A-C). The total % of spines invaded across all cells treated with Gly-0Mg^2+^ in the presence of nocodazole was 4%. Cells treated with nocodazole alone (no NMDAR activation) showed an equivalent loss of basal MT-spine invasions (data not shown). These results demonstrate that MT dynamics (polymerization and depolymerization) play a key role in basal and NMDAR-dependent spine invasions, and that synaptic NMDARs regulate MT entry into dendritic spines.

### MTs promote NMDAR-dependent structural change in targeted spines

**Figure 3:**
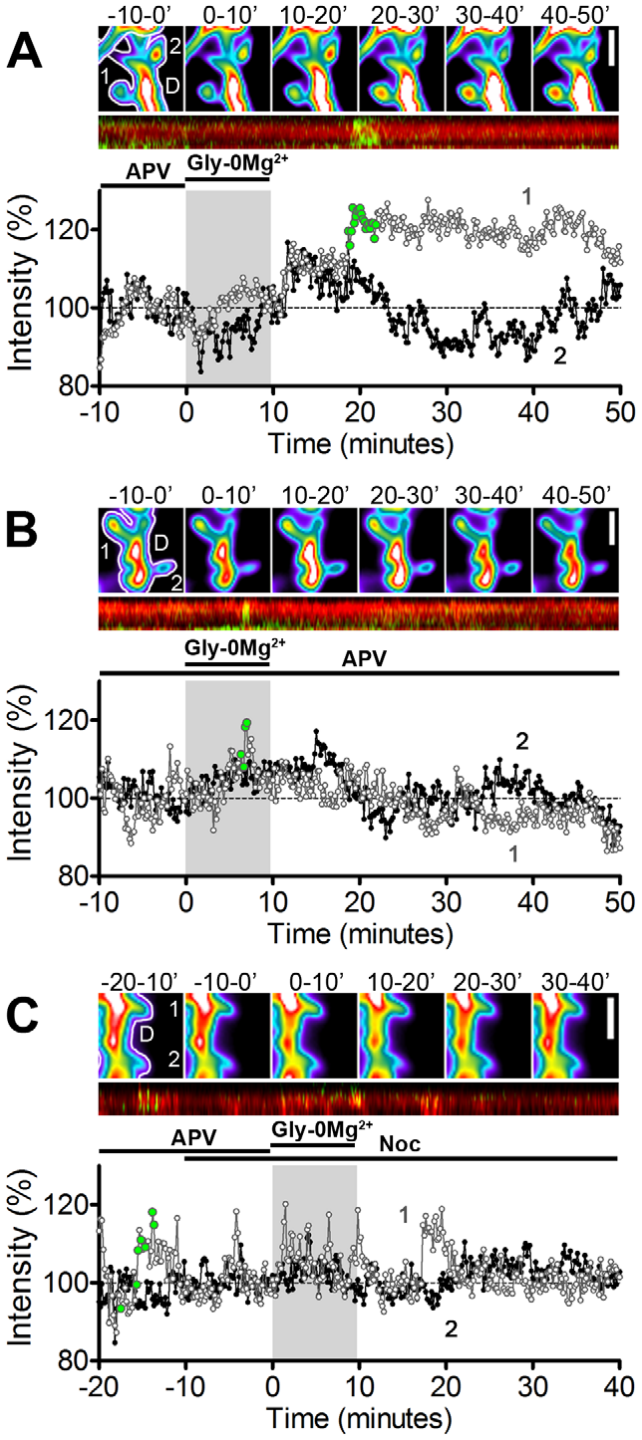
Enlargement of MT-invaded spines following acute activation of synaptic NMDARs. (A - C) Representative invaded and non-invaded spines from cells treated with Gly-0Mg^2+^ alone (A), or with Gly-0Mg^2+^ in the presence of APV (B) or nocodazole (C). *Top*, pseudocolored images of the DsRed2 signal intensity averaged over 10 minute time intervals spanning the time-lapse (i.e. before, during and after treatment with Gly-0Mg^2+^). In the left-most panel the dendrite and spines are outlined in white and are labeled (1 = invaded spine, 2 = non-invaded spine, D = dendrite shaft). Scale bars, 2 µm. *Middle*, kymographs for the invaded spines from the panels above show the timing of MT invasions during the time-lapse (EGFP-α-tubulin in green, DsRed2 in red). *Bottom*, normalized DsRed2 fluorescence intensities of the spines shown above at each frame in the time-lapse (10sec intervals; grey circles  =  invaded spines, filled black circles  =  non-invaded spines). Experimental paradigms are shown above the plot, as in Figure 1. Light grey region from 0–10 minutes indicates the timing of the Gly-0Mg^2+^ treatment. Green-filled circles indicate the frames in which the spine was invaded by a MT (also shown in the kymographs above).

**Figure 4:**
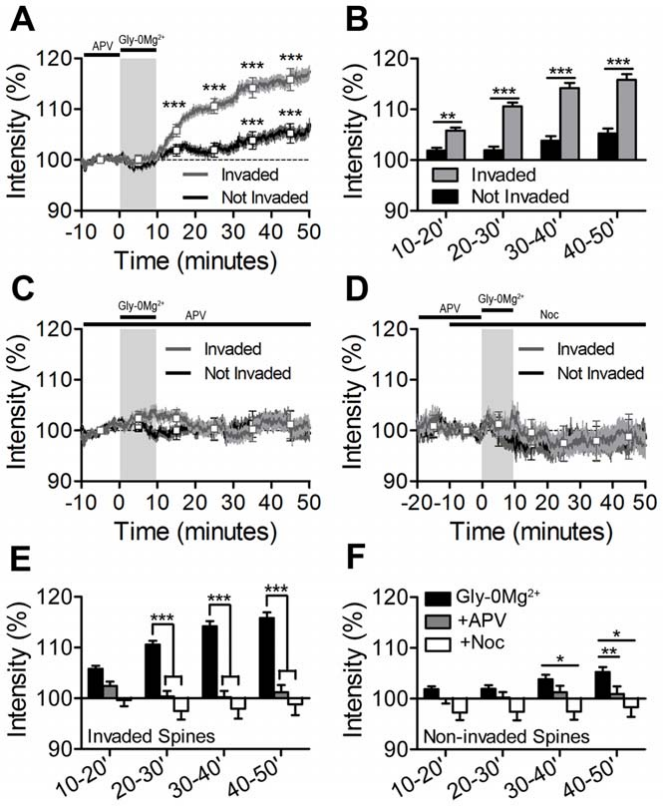
MTs are important for lasting NMDAR-dependent spine enlargement. (A) Normalized DsRed2 fluorescence intensities at each frame, averaged across all invaded (grey) and non-invaded control (black) spines from cells treated with Gly-0Mg^2+^ (mean ± SEM). Boxes represent 10-minute time averages of the respective traces (mean ±95%CI). Effects of time and spine-type (invaded vs. non-invaded) were assessed with a two-way ANOVA with repeated measures and a Bonferonni post-test to compare time columns. Experimental paradigms are shown above the plot, as in Figures 1 and 3. (B) Comparison of invaded (grey) and non-invaded (black) spine intensities in each time column after treatment with Gly-0Mg^2+^ (two-way ANOVA from (A) with Bonferonni post-test to compare invaded and non-invaded spines at each time-point) (mean ± SEM). (C - D) Normalized DsRed2 fluorescence intensities averaged across all invaded and non-invaded spines from cells treated with Gly-0Mg^2+^ in the presence of APV (C) or nocodazole (D). No significant differences were detected (two-way ANOVA with repeated measures). (E - F) Between-groups comparison of changes in DsRed2 intensity observed in invaded (E) and non-invaded (F) spines. Two-way ANOVA with repeated measures and Bonferroni post-test to compare cells treated with Gly-0Mg^2+^ alone, Gly-0Mg^2+^+APV, and Gly-0Mg^2+^+nocodazole at each time column (mean ± SEM). For all graphs, *p<0.05, **p<0.01, and *** p<0.001.

Acute chemical activation of synaptic NMDARs has been shown to induce spine enlargement, AMPA receptor trafficking, and post-synaptic LTP [15], [16], [17]. To determine whether MT invasions might contribute to NMDAR-dependent spine enlargement, we measured changes in DsRed2 fluorescence intensity to track changes in size (relative to baseline) of both MT-invaded spines and adjacent non-invaded spines over the course of each experiment [18]. Synaptic NMDAR activation produced lasting enlargement in spines invaded by MTs, beginning in the first 10 minutes following NMDAR activation and persisting to the end of the time-lapse 40 minutes later (n = 211 invaded spines, 9 cells, 5 preparations) (Fig. 3A, 4A,B). MT-invaded spines showed significant enlargement starting at t = 10–20 minutes (immediately after NMDA activation) and spine size increased throughout the imaging period, with a maximum increase of 15.83±0.08% (mean ± SEM) at t = 40–50 minutes (Fig. 4A,B). In contrast, non-invaded spines only showed significant enlargement at t = 30–50 minutes following synaptic NMDAR activation, with a maximum increase of 5.25±0.07% (mean ± SEM) at t = 40–50 minutes (n = 211 non-invaded spines, 9 cells, 5 preparations) (Fig. 3A, 4A,B). Thus, spines invaded by MTs after synaptic NMDAR activation enlarge on average ∼20 minutes faster and three times more than non-invaded spines.

Spines maintained in APV showed no significant change in size during the experiment (n = 140 invaded spines, 9 cells, 4 separate cultures) (Fig. 3B, 4C,E,F), indicating NMDAR activation is necessary for spine enlargement. Importantly, spines pretreated with nocodazole to block MT dynamics did not show changes in basal spine size, but failed to exhibit NMDAR-dependent enlargement (n = 32 spines, 6 cells, 4 preparations) (Fig. 3C, 4D-F), suggesting that dynamic MTs are required for NMDAR-dependent spine enlargement but not for maintenance of basal spine structure during the 1 hour treatment period. Treatment with nocodazole alone (no NMDAR activation) or APV withdrawal alone (no Gly-0Mg^2+^) also produced no significant changes in spine size (data not shown). Across-groups analysis of invaded (Fig. 4E) and non-invaded (Fig. 4F) spines confirmed that spine enlargement following Gly-0Mg^2+^ depends on both NMDAR activation and MT dynamics. Together, these results demonstrate that MT-spine invasions promote NMDAR-dependent spine enlargement.

**Figure 5:**
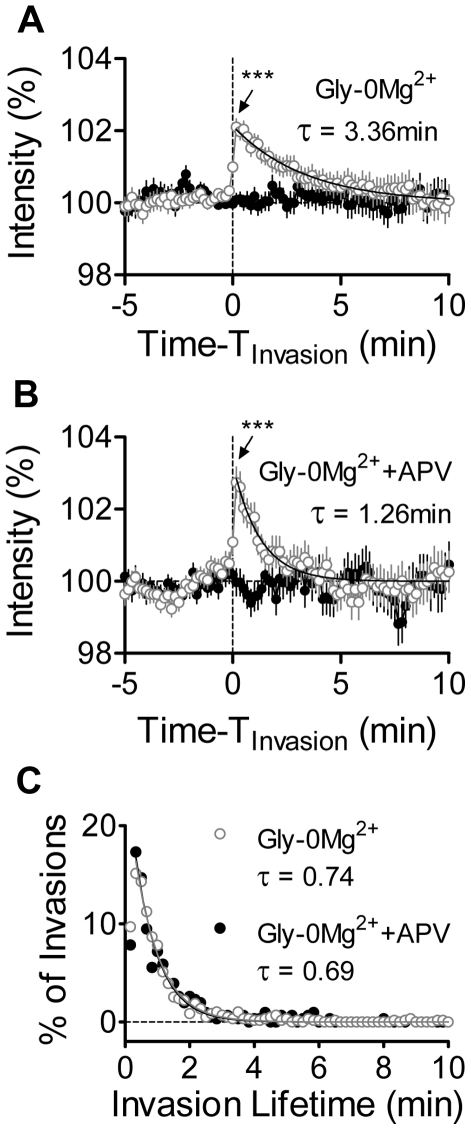
MT invasions trigger rapid spine enlargement that is more persistent following NMDAR activation. (A) MT-triggered average of normalized DsRed2 fluorescence intensities across all invaded spines (grey lines and symbols, n = 566 invasions) and non-invaded control spines (black lines and symbols, n = 566 invasions) from cells treated with Gly-0Mg^2+^. MT invasion onsets are aligned at t = 0. *** p<0.001; t-test comparing spine size one frame before (t =  −10 sec) with one frame after (t = 10 sec) invasion onset. Return to basal size was fit with a mono-exponential decay function from t = 10 sec to t = 10 min. (B) MT-triggered average of normalized DsRed2 fluorescence intensities across all invaded (n = 355) and non-invaded (n = 355) spines treated with Gly-0Mg^2+^ in the presence of APV. Symbols and analysis as in (A). (C) Population distribution of all MT lifetimes following treatment with Gly-0Mg^2+^ alone (grey open circles) or Gly-0Mg^2+^+APV (black filled circles). Best-fitting mono-exponential decay functions of invasion lifetimes for the two conditions are overlaid (grey and black lines).

In many cases MT invasions were accompanied by rapid spine enlargement (see examples in Fig. 3). To quantify these transient enlargement events, we used a “MT invasion-triggered averaging” approach to examine the short-term changes occurring in spines immediately before and after MT invasions. In this approach, the relative change in spine size occurring before, during and after each MT invasion was averaged over all invasions, with all pre-invasion spine intensities renormalized to a baseline of 100% (see Materials and Methods for details). On average, each invasion triggered a rapid increase in the size of spines treated with Gly-0Mg^2+^ alone (1.86±0.01% increase, n = 566 invasions; Fig. 5A) and in spines maintained in APV during Gly-0Mg^2+^ treatment (2.23±0.02% increase, n = 355 invasions; Fig. 5B). Peak increases did not differ between MT invaded spines treated with Gly-0Mg^2+^ alone versus Gly-0Mg^2+^+APV (two-way ANOVA), and no increase was detected in spines that were not targeted by MTs (Fig. 5A,B). These changes in intensity were not a product of spectral leakage from green to red channels upon entry of MTs because neurons transfected with EGFP-α-tubulin alone showed no increase in the red channel intensity upon MT invasion (data not shown). Importantly, the duration of the increase in spine size differed between the two groups, with spines maintained in APV returning to basal intensity ∼2.7 times faster than spines treated with Gly-0Mg^2+^ alone (Fig. 5A,B), even though the invasion lifetimes of MTs in the two conditions were indistinguishable from one another (Fig. 5C). Thus, MTs that enter spines following NMDAR activation produce longer lasting spine enlargement than invasions occurring in the absence of NMDAR signaling, irrespective of MT invasion lifetimes.

## Discussion

Over several decades the lack of evidence for MTs in spines led to an assumption that MTs, although highly concentrated in the dendritic shaft, do not extend into actin-rich spines. However, recent studies using high-resolution fluorescence time-lapse microscopy have found that MTs enter spines in every type of neuron examined to date, including hippocampal [9], [10], [11], cortical [10] and Purkinje [12] neurons. Here we document for the first time that the frequency of MT polymerization into spines increases after activation of synaptic NMDARs and that NMDAR-dependent spine enlargement is dramatically enhanced in spines targeted by MTs. This enlargement of MT-targeted spines precedes the more modest enlargement of neighboring, non-targeted spines by 20 minutes. We also demonstrate that spine enlargement events triggered by individual MT invasions after NMDAR activation persist almost three times longer than those observed during pharmacological blockade of NMDARs. Taken together, these data suggest that after NMDAR activation, an increase both in MT invasion frequency and in the persistence of spine enlargement associated with each invasion have a cumulative effect on spine size which is absent from non-invaded spines.

Activation of synaptic NMDARs with glycine and 0 mM Mg^2+^ is an established method for triggering “chemical” long-term synaptic potentiation (LTP), resulting in spine enlargement, increased AMPA receptor trafficking into the synaptic cleft and a lasting increase in excitatory post-synaptic currents [15], [16]. During induction of LTP, synaptic NMDARs open, resulting in synaptic calcium influx and activation of calcium-dependent signal transduction cascades that activate transcription, translation, and transport of synaptic molecules into spines, as well as actin polymerization and subsequent spine enlargement [19], [20]. Increases in spine size usually correlate with increases in synaptic strength following LTP ([21], but see [22]). Since increases in spine size are known to depend on actin polymerization [8], and MT and actin dynamics are intertwined in many, if not all, cell types [23], it is perhaps not surprising that MT invasions of spines contribute to spine enlargement, and plausible that they might be directly involved in LTP.

Importantly, inhibition of MT dynamics with a low concentration of nocodazole, which markedly inhibits MT invasion of spines without depolymerizing MTs, abolished the increase in spine size that otherwise reliably followed synaptic NMDAR activation. One caveat of this experiment is that MT dynamics were inhibited not only in spines but throughout both the pre- and postsynaptic neurons. However, at this concentration nocodazole did not cause changes in spine or dendrite morphology during the experiments, suggesting that it disrupted NMDAR-dependent spine enlargement without having more general deleterious effects on spines or dendrites. Moreover, 50 times higher concentrations of nocodazole do not affect presynaptic release from cultured hippocampal neurons [24].

Careful analysis of our data revealed that MT invasions occurring after synaptic NMDAR activation resulted in more persistent spine enlargement compared to stochastic invasions that occurred in cells maintained in APV. Thus, NMDAR-triggered MT invasions produce quantitatively distinct changes in spine morphology that may reflect fundamental differences in the functions MTs carry out in spines undergoing plasticity. This could reflect differences in the cargo that are transported by MTs following induction of plasticity. For example, the RhoGEF protein GEF-H1/Lfc, which is normally inactive when it is bound to MTs, is activated when it is released from depolymerizing MTs [25], and enters spines in response to depolarization [26]. Furthermore, GEF-H1 has been shown to form a complex with AMPA receptors [27], which are also transported into the spine during LTP. Another recent study suggested that the NMDA receptor subunit NR2B is transported along dendritic MTs and enters spines in response to calcium-dependent activation of CaMKII and subsequent phosphorylation of the kinesin motor KIF17 [28]. Thus, MTs entering spines undergoing NMDAR-dependent plasticity could transport select cargo to reinforce spine enlargement, resulting in the long-lasting spine enlargement that we document here.

In a previous study we found that a less specific treatment—transient neuronal depolarization with KCl—increased the frequency of MT invasions roughly two-fold [10]. Here we find that the percent of spines invaded by MTs increases roughly three-fold following synaptic NMDAR activation, from 0.65% (in the absence of NMDAR signaling) to 1.99%. However, KCl treatment also increased MT invasion lifetimes 2.5 fold [10], whereas synaptic NMDAR activation did not significantly alter invasion lifetimes. At present, it is unclear what information is encoded in the frequency and lifetimes of MT invasions of spines. If MTs are transporting cargo required for synaptic plasticity, then increased frequency of invasions may allow delivery of more cargo to an individual spine [13]. Intriguingly, a recent study showed that inducing chemical LTD resulted in dynein-mediated transport of Neuroligin 1/PSD-95 complexes out of spines [29]. It is likely that these proteins are being transported along microtubules, but this has yet to be shown directly. Thus, LTP may increase invasion frequency of MTs into spines, while LTD may decrease MT invasion frequency and/or increase MT lifetimes.

Recently a study was published showing that chemical long-term depression (cLTD) decreases MT dynamics in the dendrite shaft and the frequency of MT-spine invasions [30]. These results are consistent with those presented here insofar as they show bath application of NMDA, causing cLTD [31], [32], decreases MT invasion frequency, while we show activation of synaptic NMDA receptors, using a protocol similar to published reports that induce cLTP, increases MT invasion frequency of dendritic spines. However, it is still unclear whether MT invasion lifetimes change with LTD, as Kapitein *et al*. primarily imaged labeled EB3 puncta, which disappear once MTs stop polymerizing, making it impossible to know whether a MT has depolymerized or simply paused in the spine.

In summary, here we demonstrate that activation of synaptic NMDARs promotes MT polymerization into dendritic spines in hippocampal neurons, that MT invasions trigger rapid spine enlargement, and that MT-targeted spines show enhanced NMDAR-dependent enlargement. These findings suggest that dynamic MT entry into spines may play a role in synaptic plasticity, learning and memory.

## Materials and Methods

### Cell Culture and Transfection

All mouse procedures were approved by the University of Wisconsin Committee on Animal Care and were in accordance with NIH guidelines (university assurance number A3368–01; animal care protocol number M02130). E15.5 hippocampal neuron cultures were prepared from Swiss Webster mice of either sex (Taconic) essentially as described [33]. Dissociated neurons were resuspended in Nucleofector solution (Mouse Neuron Kit, Lonza) and transfected with human EGFP-α-tubulin and DsRed2 (Clontech) in pCAX vectors [34]. Transfected neurons were plated at low density (5×10^3^ neurons/cm^2^) on 1.0 mg/ml poly-D-lysine (Sigma)-coated glass coverslips adhered to the bottom of 35 mm plastic culture dishes that had a 15 mm hole drilled through the bottom of the chamber. Astroglial cultures from P1–3 Swiss Webster mice were plated on a separate coverslip and placed directly over the neuronal culture in an inverted ‘sandwich’ configuration to maintain robust low density neuronal cultures [35].

### Live-cell TIRF Imaging

Imaging was performed under TIRF illumination essentially as described in [10] except an Evolve EMCCD camera (Photometrics) was used instead of a Coolsnap HQ2 camera (Photometrics).

### Experimental Activation of Synaptic NMDARs

Experiments were performed on 20–27DIV hippocampal cultures. 16–24 hours before imaging, 200 µM D,L-APV was added to dishes to block NMDARs. <30 min before imaging, cells were transferred from SFM to extracellular solution (ECS): 200 µM D,L-APV in 140 mM NaCl, 5 mM KCl, 2 mM CaCl_2_, 2 mM MgCl_2_, 5 mM HEPES, and 20 mM glucose (315 mOsm). ECS was perfused through Teflon tubing at a constant rate (0.5 ml/min) by syringe pumps (New Era Pump Systems, Inc., Kent Scientific Corp.). A custom silicone insert with inlet and outlet holes was placed in the culture dish to reduce volume and control flow through the dish. Activation of synaptic NMDARs was achieved by switching the perfusion input from ECS+APV to modified ECS containing 200 µM glycine, 1 µM strychnine, 0 mM MgCl_2_, and 4 mM CaCl_2_ (Gly-0Mg^2+^ ECS) [15], [16], [17]. Following a 10 minute exposure to Gly-0Mg^2+^ ECS, the perfusion input was switched to normal ECS without any drug. When appropriate, cells were maintained in APV (200 µM) or nocodazole (200 nM) (see Results).

### Analysis of Microtubule Dynamics

Time-lapse images were acquired at 10 sec intervals for 60–70 minutes, resulting in image stacks of 361–421 frames each. Drift artifacts were corrected using the Image Stabilizer macro for ImageJ (Kang Li, http://www.cs.cmu.edu/~kangli/code/Image_Stabilizer.html). Throughout this study, spines are defined as dendritic protrusions having length ≤5 µm, and head-width >neck-width for protrusions with length >2 µm. MT-spine invasions were identified in Metamorph (Molecular Devices) by visual inspection of EGFP-α-tubulin time-lapse sequences. Kymographs were created from lines drawn along the length of invaded spines, and used to manually log the exact timing and duration of all MT-spine invasions. From these data we computed invasion frequencies, invasion lifetimes, and the percent of spines occupied in MATLAB (The Mathworks). Invasion frequency, defined as the number of MT invasions per spine per hour, was computed for each cell by counting the total number of MT-spine invasions during a time window of interest (*e.g.* from 10 to 20 minutes after Gly-0Mg^2+^ treatment), and dividing this total by the number of spines in the field of view (FOV) and by the amount of time considered. Invasion lifetimes, defined as the amount of time that MTs remain in spines after they have entered, were compared between experimental groups and time-points by pooling all invasions from each group at each time-point and computing their mean, standard deviation, and n values. Percent of spines occupied, defined as the percent of spines in the FOV occupied by a MT at any given time, was computed for every frame of each cell's time-lapse and time-averaged to compare appropriate time-windows within and across experimental groups.

### Analysis of Spine Morphological Plasticity

Spine morphology analysis was performed in Metamorph. To quantify changes in spine size during the course of each experiment, a region of interest (ROI) was drawn around each spine that was invaded by a MT during the experiment and a second ROI was drawn around a neighboring, non-invaded spine. Non-invaded spines were <20 µm from invaded spines and were of comparable size and shape to invaded spines. To adjust for background fluorescence, another ROI was drawn around a region of background near each spine-pair. DsRed2 fluorescence intensity was recorded and logged for each ROI at every time-point, and imported into MATLAB for subsequent analysis. In MATLAB, local background fluorescence was subtracted from the fluorescence intensity of each spine's ROI at every frame, after which each spine's fluorescence intensity was normalized to its own baseline value (defined as the average value over the 10 minutes prior to treatment with Gly-0Mg^2+^ ECS).

To characterize changes in spine morphology occurring immediately before and after MT invasions, “MT-triggered averages” of fluorescence intensity were generated for all invasions in a given experimental condition in MATLAB. In this analysis, for every MT-spine invasion that occurred, the time of initial MT entry into the spine was set to t = 0, and changes in the spine's fluorescence intensity occurring before and after t = 0 were recorded, so that a population summary of morphological changes associated with MT invasions could be obtained by pooling the data from all invasions of all spines in the condition. MT-triggered averages were renormalized so that the average intensity was 100% over the 5 minutes immediately preceding invasion onset and linear trends preceding invasion onset were removed by subtracting the slope of the pre-invasion average (least-squares fit).

### Statistics and Graphing

All statistical tests and graphing were performed with GraphPad Prism. Statistical tests used and test results are indicated in figures and figure legends (see Results).
